# A comparative analysis of hazard-prone housing acquisition programs in US and New Zealand communities

**DOI:** 10.1007/s13412-021-00689-y

**Published:** 2021-04-30

**Authors:** Gavin Smith, Wendy Saunders, Olivia Vila, Samata Gyawali, Samiksha Bhattarai, Eliza Lawdley

**Affiliations:** 1 Department of Landscape Architecture and Environmental Planning, North Carolina State University College of Design, Campus Box 7701, Raleigh, NC 27675 USA; 2 Earthquake Commission, Risk Reduction and Resilience, Level 11 Majestic Tower, Willis Street, Wellington, New Zealand; 3 Department of Parks, Recreation & Tourism Management, North Carolina State University, Raleigh, NC USA; 4 Department of Landscape Architecture and Environmental Planning, North Carolina State University, Raleigh, NC USA

**Keywords:** Buyouts, Hazard mitigation, Public policy, International lesson drawing

## Abstract

This article describes the preliminary results of an international comparative assessment of hazard-prone housing acquisition programs (buyouts) undertaken in six US and New Zealand communities. Semi-structured interviews were conducted with government officials and consultants tasked with administering buyout programs following flood, debris flow, and earthquake-based disasters. Key issues analyzed include local capacity, public participation, planning and design, program complexity, funding and financial management, and lesson drawing. The findings are timely given the rise in disaster-related losses, buyouts are increasingly cited as a strategy to reduce natural hazard risk and advance climate change adaptation, and national buyout programs are evolving in both countries to tackle the challenges associated with this complex process.

## Introduction

This article describes a comparative assessment of the acquisition of hazard-prone housing in United States (US) and New Zealand communities.^1^ Often referred to as buyouts, this hazard mitigation or risk reduction technique is among the most effective ways to reduce future losses when compared to other approaches like the elevation or retrofitting of structures. Buyouts typically involve the purchase of structures and land, the demolition or relocation of the housing unit, and the conversion of the land to open space in perpetuity.^2^

However, buyouts are fraught with several challenges. These include reconciling place attachment and risk reduction issues among homeowners in an empathetic manner; balancing program complexity and the timely delivery of buyout projects; developing appropriate compensation strategies based on a sound understanding of a homeowners financial standing; fostering an inclusive decision-making strategy that recognizes the contentious nature of the process and potential power imbalances between government officials and applicants; addressing concerns among local officials and residents about the loss of tax base and sense of community; and managing the resulting open space once homes are demolished or relocated.^3^ Further compounding these challenges is that few communities plan for these largely predictable situations. Since most buyouts follow disasters, there is pressure to act quickly, which can limit the active involvement of potential applicants and the larger community and hinder a local government’s ability to invest the time required to connect buyout programs with other community goals.

The results of this study are timely. The US Federal Emergency Management Agency (FEMA) is initiating the new pre-disaster hazard mitigation grant program titled Building Resilient Infrastructure and Communities (BRIC) that can fund buyout projects before a disaster strikes, and New Zealand is exploring the development of the proposed “Managed Retreat and Climate Change Adaptation Act,” which, if implemented, will create the nation’s first grant program explicitly dedicated to the acquisition of hazard-prone housing.^4^

## Study design and methods

The primary data collection technique used in this study involved semi-structured interviews conducted with local officials and consultants responsible for the management of hazard-prone housing acquisition programs in six US and New Zealand communities. The interview instrument and its associated themes were developed by drawing on academic literature, practice-based documents, and the personal experience of the two principal researchers who have led and assisted in large-scale buyout programs. Five interviews were audio-recorded, and one interview was captured using typed notes at the request of those being interviewed. The interviews were coded to identify quotes related to the content of the themes identified. Data drawn from the review of government documents and participant observations were used to reduce bias through the triangulation of data as Yin (2009) suggests.

### Site selection and data collection

Communities were selected based on a set of national and local conditions. National conditions included: (1) both nations possess mature disaster management programs; 2) both nations possess somewhat similar, yet sufficiently different, government structures (federal republic, constitutional monarchy); (3) both nations face significant hazard exposure; and (4) both nations have developed differing approaches to buyouts that are currently evolving. Local conditions included: (1) varied levels of experience with buyouts and the capacity to implement them; 2) the adoption of proactive and reactive buyout strategies (i.e., pre- or post-disaster); (3) a reliance on national, sub-national, and/or local funding mechanisms; and (4) differing hazard threats, including flood, earthquake, and debris flow. The combined characteristics of the study sites help to unpack key issues tied to the buyout process, further our growing understanding of the buyout process, and facilitate cross-cultural lessons that could be adopted by those charged with new hazard-prone housing acquisition programs, including those tied to climate change-induced retreat.

The semi-structured interviews address themes that underpin key challenges associated with buyouts. These include: (1) local capacity, (2) public participation, (3) planning and design, (4) program complexity, (5) funding and financial management, and (6) lesson drawing.

## Comparative analysis and results

Next, the literature is summarized, and the responses of those interviewed are discussed, spanning the six themes noted above.

### Local capacity

Understanding the capacity of local governments to implement buyout projects is vitally important. In their book addressing intergovernmental implementation of disaster policy, May and Williams (1986) define capacity as “the ability to reach a goal, as reflected by available resources, and by political, managerial, and technical competence” (p.28). Reflecting on the relationship between intergovernmental implementation and local capacity requires assessing how national, state (or regional in the case of New Zealand), and local governments interact. It also requires gaining a detailed understanding of the capacity of individual buyout applicants and the members of the community to participate in this process.

Past research has shown that successful buyout efforts often rely on shared governance-based strategies to bolster local capacity (Perry and Lindell 1997; Patton and Chakos 2009), to include the role of political leadership at varied levels of government (Hanna et al. 2018; Sipe and Vella 2014; Smith et al. 2018). Researchers have also shown that the ability of sub-national actors to adopt risk reduction efforts and deliver capacity-building programs that support local governments and individuals is highly variable (Burby and May 1997; Smith et al. 2013, 2017; Smith and Vila 2020). In the USA, the capacity to apply for FEMA grants that fund buyouts is particularly challenging in low wealth and smaller communities (Straub et al. 2020; Mach et al. 2019; Ross and Clay 2018; Smith and Vila 2020). In New Zealand, the capacity to implement buyouts is constrained by the lack of national support to build the local capacity required to implement housing acquisition projects (Hanna et al. 2018; Saunders and Smith 2020). Hanna et al. (2020) found that buyouts in New Zealand can be professionally, politically, financially, culturally, and socially challenging, as the necessary intergovernmental frameworks and resources are seldom in place to support effective, equitable, responsive, and robust decision-making.

When asked about the types of resources states can provide, such as data, existing policy guidance, or training relative to buyouts, one US community official said,

Well, I’ll say that we get very little help with that from the state. They’re more interested in making sure that we follow whatever rules FEMA has.

This points to an ongoing dilemma with US buyouts, which is the focus on compliance with narrowly defined, highly prescriptive federal program rules rather than the widespread provision of training and educational programs targeting locally specific capacity building needs (Smith 2011; Smith and Vila 2020).

A closely related post-disaster condition that shapes the ability to provide needed assistance to local governments in the USA is the rotation of federal and state staff during a disaster who often possess differing interpretations of national buyout policy (Smith 2011, p. 37). This can lead to slowdowns in the process and breakdowns in the level of trust (de Vries and Frasier 2012). According to one local official in the USA:

[the state] …has had a constant turnover of people. And every time they bring in new people, we have to answer the same questions, submit the same paperwork, do the same work all over again. And sometimes the next person coming in has a different spin on it, asking for additional information. So they keep us in a state of flux all the time.

In the USA, it is common for experienced governmental officials to leave their jobs for better paying private sector employment, which can reduce governmental capacity, unless local and state governments hire them as consultants (Smith 2009, p. 232; Smith 2011, pp. 46-47). The shift in capacity across institutions (i.e., public to private) can prove challenging following disasters as the number of consultants available to assist local governments may not be able to meet local and regional demands. As noted by one US community official, they struggled to find qualified consultants, whereas another larger, wealthier jurisdiction that was interviewed has hired a team of on-call consultants with “experience in other parts of the country with voluntary buyouts [in order] to leverage their expertise.”

In New Zealand, multiple respondents noted the importance of retaining staff across the buyout process, which facilitated consistency and the building of trust. The research team did not, however, glean from New Zealand officials whether they believe that there is an adequate number of staff experienced in the management of buyouts to address the demands associated with future buyouts, including those tied to the possible operationalization of the proposed Managed Retreat and Climate Change Adaptation Act.

The limited number of officials experienced in the management of buyouts in New Zealand is less extensive when compared to the USA. This is due to the smaller number of buyouts that have occurred in New Zealand communities. In addition, there are no national programs that explicitly target the buyout of hazard-prone properties, and as a result, there is less demand for this type of expertise until an event occurs (Glavovic et al. 2010). For instance, in response to the 2010 Darfield Earthquake, the New Zealand Parliament adopted the Canterbury Earthquake Recovery Act of 2011, established the quasi-governmental Canterbury Earthquake Recovery Authority (CERA) to lead recovery efforts, and allocated the funding used to purchase over 8000 properties (MacDonald and Carlton 2016).^5^

The “capacity” of residents to participate in a buyout is influenced by a number of factors. These include determining the ability of applicants to cover some or all grant matching funds (applicable in some US jurisdictions), determining if a buyout provides adequate monetary compensation given a family’s unique financial situation, and identifying alternative housing options pending a buyout offer. A closely related obstacle to a resident’s participation in a buyout program involves their ability to make temporary repairs to their home in order to make it livable during the interim period between the time a homeowner is deemed an eligible applicant and the receipt of buyout funding. Decisions must be made, for instance, whether to use personal savings or insurance proceeds (if available), seek additional grants or loans, or solicit other forms of assistance like donated building materials or the provision of volunteer labor.^6^

When discussing how to advise residents what to do during this interim period, one US official lamented that while homeowners had offers from non-profit groups to repair homes slated for buyout, those tasked with buyout administration discouraged non-profits to make temporary repairs to these structures.

…we advised them [non-profit organizations] not to work on that house …and move on to the next one. Turns out that was bad advice. But we didn’t know better, [that] they would have been better off to go ahead and repair the person’s home, allow them to live in it for four years. And they could still have taken the buyout later. They would have had a much better living situation. But that’s just something we didn’t know going into it.

Reducing the options available to buyout applicants raises questions about the voluntary nature of the buyout process and how those with less discretionary income and political influence were effectively blocked from acting in their own best interests by governmental decisions beyond their control. It also highlights the ongoing struggle to apply good governance and planning strategies needed to coordinate the timing of resource delivery at different points in the post-disaster recovery process. In this case, local officials were acting based on information provided by state officials who promised that buyout funds were being expedited.

### Public participation

In the USA, the acquisition of hazard-prone housing using federal funding prohibits local governments from employing their legal ability to condemn property for a public purpose and stipulates that any homeowner participating in the program does so voluntarily (FEMA 2015). In New Zealand, the ability to acquire hazard-prone housing is more nuanced and driven by the national legislative mechanism being implemented. This has included event-specific legislation resulting in “red-zoning” properties post-disaster to prevent future loss (Hanna et al. 2018; Saunders and Smith 2020).

Researchers have questioned the degree to which buyouts are actually “voluntary programs,” and whether the largely post-disaster driven process provides an adequate forum for public participation and debate. Important questions include whether buyouts are the preferred risk reduction option among residents, whether it is in the homeowner’s best interest to remain in the program, and whether historically marginalized populations are actively involved throughout the process (Binder and Greer 2018; Fraser et al. 2003; Elliott et al. 2020; de Vries and Frasier 2012; Baker et al. 2018; Nguyen 2020).

In New Zealand, in particular, following the Christchurch earthquake, the classification of “red-zoned” properties prone to liquefaction during seismic events negated the repair or reconstruction of damaged homes and supporting infrastructure, which limited alternatives to taking the buyout offer (Nguyen 2020; Hanna et al. 2018). The “voluntary” nature of the red zone acquisition process was raised in court proceedings, where it was held that:

It is true that the Crown did not use its powers of compulsory acquisition under the Act. However, it is unrealistic to describe the transactions that occurred as voluntary. The inhabitants of the red zones had no realistic alternative but to leave, given the damage to infrastructure and the clear message from the government that new infrastructure would not be installed, and that existing infrastructure may not be maintained and that compulsory powers of acquisition could be used.^7^

More recently, it was reported in the New Zealand media that residents involved in a buyout process undertaken in response to a debris flow “reluctantly and under duress” signed settlement agreements, despite the official court proceedings showing that those who settled had done so willingly.^8^ In both New Zealand cases, the differing interpretations among buyout administrators interviewed and buyout recipients of what constitutes a voluntary program align with prior research conducted in US communities (Greer 2015).

A significant amount of research highlights the factors that influence a residents’ decision to participate in a buyout (Binder et al. 2015; Fraser et al. 2006; Greer 2015; de Vries and Frasier 2012; Saunders and Smith 2020), which includes physical factors such as the location of homes in a floodplain (Robinson et al. 2018) and the level of damage sustained to one’s home (Myers et al. 2008; Nguyen 2020). Financial matters affecting participation include the consistent availability of funding (Boston 2019), the price offered homeowners (de Vries and Frasier 2012), the requirement of homeowners to provide matching funds (Fraser et al. 2006), and the length of time required to receive payment (Binder and Greer 2016; Spee 2008; Young 2018; Saunders and Smith 2020). Psycho-social considerations include place attachment (Binder et al. 2015; Handmer 1985; Kick et al. 2011; Henry 2013; Harker 2016), the existence of strong social ties (Shriver and Kennedy 2005; Perry and Lindell 1997), perceived risk and past disaster experience (Greer 2015; Kick et al. 2011; masked for review), and trust in the local buyout manager (de Vries and Frasier 2012).

Concerns regarding the perception of risk were expressed in New Zealand as evidenced by a local government official who noted:

Some of them [residents] don't believe the risk is there. Some don't believe that the council should be exercising its statutory responsibility in this way, forcing people out of their homes. So that's our house, our domain sort of thing, keep away.

It can be incumbent on local leaders to help reconcile reducing future hazard exposure and using the buyout to help individuals recover while recognizing the highly personal nature of the process and the reality that in some cases of extreme risk there may be few alternatives to a buyout. According to one local government official in the USA, “We have found with property owners, if up front they don’t understand the reason that we’re there and don’t understand the why behind the entire process, they’re less likely to participate.”

The participatory planning literature suggests that alternatives to traditional public meetings can provide a venue to address power imbalances and foster a more interactive dialogue (Arnstein 1969), which is particularly important when planning for buyouts (Siders 2018; Scott et al. 2020). One US community official described a different approach to public meetings:

We’ve had meetings out in the front yard with a group of a dozen houses say that we’re looking to approach and discuss a buyout with. We’ll just pull up in a truck and put the tailgate down and invite people to come out and have those conversations.

A New Zealand official similarly noted the benefits of relationship building and its linkage to a broader participatory planning process:

…one is around the relationship you build, not only with your professional colleagues, but the relationships you've built with the landowners. And in this case, I've known the landowners, the majority of them, since 2005 [planning for the buyouts began in 2016]. But I think the importance of the relationship where people are treated with respect and dignity, is fundamental to the success of the outcome. And I think having processes that are robust and transparent, are really important. And being open and communicative with people as well. And having done that, the actual delivery is, I wouldn't say it's been seamless, but it's been pretty efficient, which has been quite rewarding.

### Planning and design

Planning and design have the potential to address key challenges, including the perceived equity of buyout processes and outcomes among recipients (de Vries and Frasier 2012; Fraser et al. 2006; Scott et al. 2020), especially if residents are involved in a meaningful way (Perry and Lindell 1997). Additional benefits include reducing the loss in tax revenues if replacement housing strategies are created (Wiley 2018), injecting energy efficiency into replacement housing (Jenson and Fantle 1979), achieving broader community goals like economic development (David and Mayer 1984), restoring and connecting habitat and communities (Kihslinger and Salvesen 2017; Coastal Dynamics Design Lab 2019; Flink 2020; Conrad et al. 1998; Highfield et al. 2019), and managing post-buyout open space (Brand and Nicholson 2016; Greater Christchurch Group, Department of the Prime Minister and the Cabinet 2016).

Other hazard scholars have suggested that buyouts may not serve the best interests of communities or individual participants, citing reactive projects that have torn apart the social and physical fabric of communities or have been used to target low-income residents and minorities as a form of “disaster gentrification” (Weber and Moore 2019; Scott et al. 2020). Viewed through the lens of the political economy of development literature, planning and design efforts can be co-opted by those in positions of power, and if the views and goals of the powerful conflict with buyout participants and staff charged with program administration, buyouts can proceed with limited input from those most directly affected (Tobin and Peacock 1982). This is particularly evident in communities that do not possess an influential advocate to voice the community’s values and desires (Freudenburg et al. 2009). There is also evidence to suggest that in US tribal communities, buyout program rules undergirded by the market value of homes, individual choice versus collective action, and how property is defined may disadvantage US tribal communities and further entrench inequality (Marion 2018). The degree to which these factors as well as complicated land ownership arrangements may influence the buyout choices of the indigenous Maori in New Zealand is worthy of comparative study.

Further compounding potentially negative results is the reality that most US communities do not plan for buyouts. This in spite of the fact that such actions are encouraged by FEMA as part of a local government’s hazard mitigation planning efforts. As required under the Disaster Mitigation Act of 2000, local governments must develop a hazard mitigation plan in order to remain eligible for pre- and post-disaster hazard mitigation funding (FEMA 2015). Local official’s reluctance to identify at-risk properties and publicly disclose them in planning documents are tied to concerns that this may have a negative effect on property values, and if a buyout is implemented, will result in a loss of tax base, residents voice opposition to being bought out, or administrators believe that this legally commits the jurisdiction to take action when communities may not have the resources to do so (Smith et al. 2013). These conditions point to the disconnect among US community officials between pre-event hazard mitigation planning, design, and grants management. One local government official described the buyout process as an unforeseen event, and as a result, they did not plan for this eventuality even though they had implemented a buyout project following a previous disaster.

If you could foresee the future, and you knew that you were going to have a buyout program, and if you went to that person and said, hey we want to offer you a buyout, and here’s where you could possible go live. And here’s how we can couple some additional financial resources with the funding that you’re going to get from the buyout in order to make you whole again, is this something you’re willing to do?

Additional comments further highlight the decoupling of planning and grants management administration. As stated by one official:

It's very unlike a road project or a traditional park project, or building a school, where you can say these are the properties we need to acquire, and this is what the vision for the use of these properties is, and we're going to implement it that way. That's really not possible with voluntary flood buyouts. You have to see what you end up with at the end.

The reactionary roles assumed by US community officials are closely tied to the roles assumed by states in buyout planning and design (Smith and Vila 2020). When a local official was asked about the ability to foster a comprehensive community-wide buyout effort that reduces a common problem known as “checkerboarding,” where some property owners choose to participate and other neighbors do not, they noted that “the state made selections [eligible buyout properties] without our input, and so unfortunately, we’re going to end up with a checkerboarding effect in some of these older neighborhoods.”

New Zealand communities, which have placed an emphasis on planning for buyouts without necessarily having the funding in place, address some of the concerns found in US communities that fail to engage in proactive approaches. Following disasters, New Zealand communities have sought to align national planning mandates with the buyout process as they search for funding. And yet, because New Zealand communities do not have ready access to the funding required to implement housing acquisition programs, this has hindered pre-disaster buyout planning (Hanna et al. 2018; Boston 2019). The national and local contextual factors present in both countries suggest the need to blend certain aspects of US and New Zealand approaches, to include developing a meaningful national buyout program in New Zealand that draws from the lessons of the US Disaster Mitigation Act, including the need to more closely align pre-event hazard mitigation plans with specific projects and associated funding. The USA stands to benefit from New Zealand’s integration of plans, flexibility of buyout programs, and the widespread development of open space management strategies.

An increased, albeit varied emphasis on the application of design processes and the programming (which identifies the scope of work to be designed) of land uses following buyouts has emerged in the USA and New Zealand (Hanso and Lemanski 1995; Coastal Dynamics Lab 2019; Flink 2020; Kihslinger and Salvesen 2017; Brand and Nicholson 2016; Greater Christchurch Group, Department of the Prime Minister and the Cabinet 2016). In the case of the Christchurch buyouts, the national government took the unusual approach of appointing the national crown property agency, Land Information New Zealand (LINZ), to manage the acquired red zoned flat land. This task was different than their typical land management responsibilities which focus on the oversight of pastoral land where farmers lease the property for grazing and cultivation. In the case of the Christchurch buyouts, LINZ did not provide an option to lease back land that was bought out but rather played an important role in the Christchurch open space planning process:

LINZ had the experience in managing Crown [national] land, so [while] we had structures in place, [the Christchurch case represented] a different portfolio compared to high [mountain] and back country land. [Regeneration plans] provide a once in a lifetime opportunity to shape the future of the city, and [the plans can be] used for education, cultural development, tourism, and encouraging indigenous flora and fauna.

Regeneration plans, created to guide the use of the resulting open space post-buyout, were developed by the council, iwi (largest social unit in Maori society), and communities. Once completed, the land is transferred back to local councils. In the Waimakariri District (a neighboring council which suffered significant earthquake damage), local officials completed a plan which includes proposed projects such as a BMX track, dog park, “food forest,” river walk, restored forest, and ball fields (Waimakariri District Council 2020).

Open space management in New Zealand is ultimately managed by territorial authorities through reserve management plans as required in the Reserves Act of 1977. Under this Act, councils are required to prepare and develop management plans for reserves under their control. The purpose of a reserve management plan is to:

...provide for and ensure the use, enjoyment, maintenance protection and preservation, as the case may require, and to the extent that the administering body’s resources permit, the development as appropriate, of the reserve for the purposes of which it is classified... (Reserves Act of 1977, Section 41 (3).

Reserve management plans are not simply created for reserve design purposes; they also require a set of objectives and policies through which design proposals can be critically and effectively assessed. Following the Christchurch earthquake, regeneration plans (such as the Ōtākaro Avon River Corridor Regeneration Plan) were included in the Greater Christchurch Regeneration Act of 2016. In order to comply with the stipulations of the Act, these plans were required to be consistent with plans approved under the Section 41 of the Reserves Management Act, or any other management plan for a reserve under any other legislation (Section 63 Greater Christchurch Regeneration Act 2016).

In the USA, the management of buyout-related open space, which also falls to local governments or other supporting organizations, is not guided by national planning policy. Nor is there clear guidance provided by funding agencies, other than the severe restrictions placed on the land’s use (Zavar 2016). Further compounding the lack of policy or design-based guidance is that many communities do not plan for the management of open space, especially those without the staff, funding or technical expertise required to develop and implement plans, to include managing the land effectively over time. As a consequence, buyouts often result in a dispersed pattern of vacant lots rather than well-designed public spaces (Zavar and Hagelman 2016). One local official in the USA stated: “…there’s not been very much conversation [about open space management] nor has there been any real guidance other than what you can’t do.” This quote highlights the reality that local officials and their contractors are often so focused on grant program administration that either they do not have the time or have not been asked to think through how their work connects to broader community issues, including the creation of public spaces.^9^

### Program complexity

Buyout programs in the USA and New Zealand are notoriously complex and time-consuming. For instance, an acquisition process involving the resettlement of Allenville, Arizona, 33 federal, state, and local entities were involved in the process, which significantly impeded what was ultimately considered a successful effort (Perry and Mushkatel 1984). Further complicating the buyout process are the myriad rules varied organizations employ that are tied to project eligibility and implementation, to include how these rules are subject to change over time. Changes in project rules can occur due to access to new information by administrators, the involvement of government officials who interpret rules differently, or in the case of New Zealand, the adaptation of an existing legislative mechanism (or post-disaster creation of new ones), and the integration of buyouts with local planning requirements.

Perhaps the clearest manifestation of the complexities associated with buyouts is the length of time it takes to complete the process. In a US study led by the Natural Resources Defense Council, which assessed 30 years of FEMA data, they found that it took on average more than 5 years between the time a flood occurred and the completion of a project (Weber and Moore 2019). In New Zealand, it has taken more than 15 years to find the funding and develop the planning framework required to implement a buyout program in one community, during which time many residents were allowed to rebuild in situ before a program was developed (Hanna et al. 2018). One New Zealand official tasked with managing the buyout program in this community noted that …“the government wouldn’t support managed retreat unless [a] legal framework [was] there, hence the plan change.” This required rezoning the land and amending their regional plan to ensure that local and regional council policies were aligned.

Research conducted by New Zealand scholars suggests that future acquisition programs should employ robust governance strategies that reduce complexity across different property acquisition mechanisms and provide a nationally consistent and transparent allocation of decision-rights across the tiers of government (Boston and Lawrence 2017; Lawrence et al. 2020). The ability to balance governance strategies that are undergirded by existing planning laws points to the value of pre-event planning for buyouts and managed retreat in order to facilitate plan consistency in advance. This allows for the implementation of the buyout process in a more timely and inclusive manner.

Based on preliminary observations associated with this research, it appears that New Zealand’s option to choose the most appropriate legislative mechanism for the local situation at hand, and the flexibility to develop a disaster-specific buyout framework, can speed up the buyout process once funding is available and planning requirements are met. This is consistent with disaster recovery planning research, which suggests that taking the time to slow down and assess the situation, initiate planning processes, and implement collaborative problem-solving measures can speed up reconstruction activities when funding arrives (Chandrasekhar et al 2014). The lengthy post-disaster planning horizon (15 years) described by one New Zealand official highlights challenges both councils and communities face when managing the uncertainty of information, external decision-making, and funding.

In the USA, programs and policies explicitly tied to buyouts include a well-established set of pre- and post-disaster hazard mitigation funds that follow a more routinized, albeit complex, set of rules that do not necessarily reflect local conditions and needs. These conditions can lead to lengthy implementation timelines and hinder good community engagement strategies that further undermine local self-determination among residents participating in the buyout as well as efforts to achieve larger community goals (Baker et al. 2018; de Vries and Frasier 2012).

One US official noted that:

… we had some folks that took so long from when they submitted their application to when we actually got the funding that they had gone ahead and had their home repaired or rebuilt. So when it came time, they didn’t want to start over [and] they ended up withdrawing from the buyout program.

### Funding and financial management

In the USA, the micromanagement of buyout funding by states and the federal government was highlighted by multiple communities as a major impediment, citing the desire to be given greater flexibility to more readily obtain reimbursements for work performed, identify eligible buyout properties, and achieve complimentary community goals. In response to these problems, one US community developed their own locally funded buyout program to escape the rigid interpretation of federal and state rules and to better connect their buyout program to community goals. Specific goals identified in their hazard mitigation plan include improving the water quality of urban streams, expanding greenways, acquiring otherwise ineligible properties, spending more time getting to know potential applicants and their unique needs, and accounting for the loss of taxable income following buyouts by developing new housing near lands converted to open space. Researchers have also devised proposed property selection criteria that place an increased value on parcels adjacent to other buyout parcels, wetlands, parks, and other open spaces to encourage more clustered buyouts (Highfield et al. 2019).

In New Zealand, there is currently no consistent or national framework for funding buyouts (Boston 2019). As a result, local governments must develop their own funding strategy, which may include drawing on rates (i.e., local taxes) via regional councils and territorial authorities, and in some cases relying on national government co-funding. This approach has the potential to create inconsistencies and inequities across the country (Boston and Lawrence 2017; Boston and Lawrence 2018).

Some New Zealand community officials interviewed have invested a significant amount of time planning for buyouts as they waited for funding, and reported better outcomes, such as the speed of implementation and the management of open space, once monies became available. A local official in New Zealand expressed a mix of enthusiasm and trepidation when describing the merits of their approach, including uncertainties regarding the availability of necessary funding.

…you’ve got to be committed, if you say you’re going to buy property when someone knocks on your door, you got to be in a position to do that. I was kind of worried that we suddenly had everyone come in, and we said we’d buy a property, but we actually can’t do that right now because we’ve already bought 20 and that’s all we can purchase right now.

One underreported financial challenge facing US communities, especially smaller and low-wealth jurisdictions, is the ability to pay for the activities required to administer the program up front, drawing from general savings or account balances and then seeking reimbursement from the state. One official noted that … “they [the state] were fortunate that we had enough fund balance … that we were financially strong enough to float the money,” whereas another smaller jurisdiction noted their concerns about seeking reimbursement, particularly given the length of time it took to obtain funding from the state once requested.

Several US and New Zealand respondents noted the benefits of additional compensation beyond the pre-disaster fair market value of properties offered by traditional national programs in both countries. One New Zealand official remarked that under the Public Works Act, residents could receive up to NZ$35,000 to vacate possession of their property on an agreed-upon date, as well as up to an additional NZ$10,000 for reaching an agreement within 6 months of the start of the negotiation process. In the USA, local government officials praised additional state-based funding to supplement federal dollars, thereby making offers more attractive to low-income homeowners.

### Lesson drawing

A number of government agencies and researchers have created “lessons-learned” documents post-buyout (Freudenburg et al. 2016; Canterbury Earthquake Recovery Authority 2016; Smithies et al. 2015). Yet, Greer and Brokopp Binder (2017) suggest that the transfer of these “lessons” to widespread practice remains insufficient. Similarly, researchers in New Zealand have expressed the need for policy learning as part of a comprehensive approach to developing a buyout program (Boston 2019). In spite of the generation of documents and recommendations, those charged with buyouts at the community level in both the USA and New Zealand expressed their frustrations regarding the ineffective transfer of lessons to both those developing and implementing a buyout program and those able to affect policy change. According to a local government contractor in the USA:

One of the other beefs we have is that there’s not enough knowledge transfer. And I’m not sure [if that’s] because the knowledge ain’t there, or it just don’t get transferred as easy to us, but we have literally had to create procedures on our own.

In another case, an exasperated New Zealand official noted that regional officials were encouraging other regional and local councils to contact them regarding lessons when in fact they had yet to receive the funding needed to begin purchasing homes. Similarly, a local government official in the USA stated: “What good does it do to give you [the interviewer] answers [lessons], how we can benefit other communities, when we can’t get our own program up and running.”

Lesson drawing also proved to be problematic at the national and state level. While lessons were captured by those involved in the Christchurch buyouts and posted to a website, the larger institutional body CERA was ultimately disbanded, and much of the information is difficult to find. In the USA, the failure to codify state-level emergent disaster recovery organizations and the programs they develop and manage (including those addressing buyouts) has led to the loss of institutional knowledge and experience. It has also hampered the ability to capture an understanding of how to reconstitute these organizations and programs following the next disaster. This can result in the unnecessary post-disaster “re-learning” of lessons in subsequent events, which ultimately hinders the effectiveness of future buyout programs (Smith 2014; Smith et al. 2018).

## Conclusions, recommendations, and future research

This article’s findings, recommendations, and future research are discussed next, drawing from key themes including local capacity, public participation, planning and design, program complexity, funding and financial management, and lesson drawing. Research assessing the local capacity needed to develop and implement buyout programs has highlighted the importance of intergovernmental coordination and governance. Assessing the relationship between governance and local capacity requires understanding more than the abilities of national, state, and regional entities to build a local government’s capacity to administer the buyout process. It also necessitates understanding the “capacity” of individuals residing in a community, including buyout recipients, to express their needs effectively and for this information to inform national, sub-national, and local actions. Unfortunately, this is not always the case as reflected in differing perceptions among government officials and potential applicants regarding what constitutes a voluntary buyout and narrowly defined buyout rules promulgated by national, state, and regional agencies that fail to account for local needs and conditions. This requires developing new or amended national and sub-national buyout policies that are more adaptive and able to account for individual and community variations in capacity and needs, including how they both change over time.

Local officials in both countries described how the capacity to address many of the challenges associated with buyouts is tied to staffing issues. US interviewees noted several problems related to the post-disaster rotation of differing federal, state, and local officials into communities dealing with buyouts. This has resulted in an uneven level of capacity, problems in retaining pertinent knowledge, and difficulties in maintaining trust between governmental actors and buyout applicants. Conversely, several New Zealand officials noted the importance and benefits of maintaining staff continuity. Given the small number of buyout projects undertaken in New Zealand, maintaining a national cadre of adequately trained officials is unlikely. However, with the emergence of a new national statute on managed retreat that may fund buyouts on a more consistent basis, it is likely that the number of trained personnel will need to increase. The cost required to support staffing and a training program could be considered in the (yet to be drafted) provisions of the proposed Managed Retreat and Climate Change Adaptation Act or through increased government funding for councils. In the USA, additional funding, perhaps provided through BRIC, could be used to fund an increased emphasis on local capacity building efforts.

Maintaining institutional continuity also proved important according to those interviewed. For example, a significant amount of knowledge and experience was gained by those involved in the Christchurch buyouts, to include how to create an institutional body charged with program administration (CERA) and the use of an existing national land management agency (LINZ) to help oversee the resulting open space generated by the buyout of thousands of homes. Yet questions surrounding whether the dissolution of CERA may hamper the institutional memory required to effectively plan for and manage future large-scale buyouts or whether LINZ has fully captured how to plan for and manage open space generated through buyouts by codifying this process in organizational structures, policy, funding, and staffing is uncertain and should be further explored.

Many of the US officials interviewed described a disconnect between planning and grants management which manifested itself in post-disaster buyout strategies driven by narrowly defined federal and state rules rather than local needs and conditions. In the USA, community officials regularly complained about the micromanagement of the buyout process by state and federal officials, which was reported to hinder flexibility and innovation. These comments were made 20 years after the passage of the Disaster Mitigation Act, which requires the development of hazard mitigation plans in order to gain access to buyout funding. Yet few communities pre-identify buyout properties in these plans nor prepare for the management of the resulting open space.

Relying on a largely post-disaster, reactionary approach to buyouts, in spite of a national planning mandate that encourages proactive behavior, highlights the need to create clearer incentives to pre-identify potential projects and plan for their implementation. One way to incentivize the pre-identification of potential buyout projects is to provide communities additional BRIC funding if hazard mitigation plans include buyout projects and allow for the more flexible use of buyout funding if an approach is clearly articulated in hazard mitigation plans that aligns a buyout project with other community plans and associated goals. In one US community studied, they created their own buyout program in order to avoid federal and state policy constraints and to better coordinate the acquisition of hazard-prone housing with other community goals found in plans, including the development of an expansive greenway system.

The USA should draw lessons from New Zealand’s approach to open space management, to include the development of regeneration plans, and to identify appropriate organizations best suited to assist states and local governments develop these plans and manage the resulting open space over time. This idea should be augmented by creating leasing agreements for uses of land beyond the narrow confines of current FEMA policy (which requires changing FEMA rules); the expansion of buyout funding mechanisms to support the purchase of contiguous parcels and those adjacent to parks, wetlands, and greenways (that may not be otherwise eligible); and the provision of funds to support the development and implementation of open space plans that include a clear approach to maintaining the land over time.

New Zealand officials noted the benefits and challenges of embedding buyouts in local and regional planning processes tied to national planning mandates. New Zealand’s complex planning regime required local officials to ensure buyouts aligned with planning laws, which slowed down the process initially. New Zealand officials acknowledged that while the use of varied national funding programs not originally focused on the buyout of hazard-prone housing provided additional flexibility to address unique local conditions, the lack of a national buyout program required communities to develop their own funding plan or wait for the appropriate funding source to be identified and allocated. According to New Zealand officials, this can lead to potential inconsistencies and, when coupled with making sure that buyout projects comply with multiple planning requirements, has led to significant delays. New Zealand officials did note that the time spent on planning for the buyout process sped up the implementation time once the funding became available, which is similar to findings in the disaster recovery literature. In order to recognize the benefits and address the constraints of this approach, local and regional councils should integrate potential buyout projects in existing plans (and those ultimately tied to the Managed Retreat and Climate Change Adaptation Act) before a disaster strikes, drawing from the US experience that demonstrates the need for a clearer nexus between planning and grants management. The following policy recommendations proposed in New Zealand should complement the development of a national program with consistent funding to pay for the cost of future buyouts identified in plans.

Addressing key challenges described in this article requires developing or amending national buyout programs and policies that reflect the diversity of communities, the presence of adequate and flexible funding mechanisms, and capacity building programs undergirded by pre-disaster planning. As the USA and New Zealand move forward with ambitious proposed and emerging national programs addressing the buyout of hazard-prone housing, the research findings, policy recommendations, and proposed future research stand to improve the ability of both countries to better address the challenges identified by those tasked with the implementation of these programs at the local level. In an era of climate change, the need to share and adopt lessons across nations to reduce future losses and adapt has never been more important.

## Footnotes

1 This material is based upon work supported by the US Department of Homeland Security under Grant Award Number 2015-ST-061-ND0001-01. The views and conclusions contained herein are those of the authors and should not be interpreted as necessarily representing the official policies, either expressed or implied, of the US Department of Homeland Security.

2 In one New Zealand community, a stopbank or levee will be built on the acquired land.

3 Concerns over the loss of tax base is primarily a US issue, although it may become more relevant in New Zealand if large-scale buyouts and managed retreat results in the movement of significant numbers of individuals to another council’s jurisdiction.

4 BRIC funding is based on 6% of the previous year’s total disaster losses across the USA. When applied to the 2020-2021 funding cycle, the total allocation approximates 500 million dollars (FEMA 2019).

5 The buyouts that occurred in the Christchurch area are the largest undertaken in the history of New Zealand and drew extensively from earthquake insurance proceeds as part of the total buyout offer.

6 Interviews conducted with US and New Zealand officials as well as the larger literature has shown that the length of time it takes for individuals to receive buyout funding is highly variable and can range from months to years after a precipitating event.

7 Quake Outcasts v The Minister for Earthquake Recovery (2015) NZSC 27, paragraph 140

8 https://www.stuff.co.nz/bay-of-plenty/300184893/coastal-residents-fight-for-their-homes-comes-to-an-end, 16th December 2020

9 In the case of the respondent cited, an open space strategy has been developed for their community as part of a multi-year, interdisciplinary effort undertaken by two nearby universities, but the contractor tasked with the administration of the buyout was not aware of this effort.
